# Discovering Preferential Patterns in Sectoral Trade Networks

**DOI:** 10.1371/journal.pone.0140951

**Published:** 2015-10-20

**Authors:** Isabella Cingolani, Carlo Piccardi, Lucia Tajoli

**Affiliations:** 1 Department of Management, Economics, and Industrial Engineering, Politecnico di Milano, Italy; 2 Department of Electronics, Information, and Bioengineering, Politecnico di Milano, Italy; Universidad Rey Juan Carlos, SPAIN

## Abstract

We analyze the patterns of import/export bilateral relations, with the aim of assessing the relevance and shape of “preferentiality” in countries’ trade decisions. Preferentiality here is defined as the tendency to concentrate trade on one or few partners. With this purpose, we adopt a systemic approach through the use of the tools of complex network analysis. In particular, we apply a pattern detection approach based on community and pseudocommunity analysis, in order to highlight the groups of countries within which most of members’ trade occur. The method is applied to two intra-industry trade networks consisting of 221 countries, relative to the low-tech “Textiles and Textile Articles” and the high-tech “Electronics” sectors for the year 2006, to look at the structure of world trade before the start of the international financial crisis. It turns out that the two networks display some similarities and some differences in preferential trade patterns: they both include few significant communities that define narrow sets of countries trading with each other as preferential destinations markets or supply sources, and they are characterized by the presence of similar hierarchical structures, led by the largest economies. But there are also distinctive features due to the characteristics of the industries examined, in which the organization of production and the destination markets are different. Overall, the extent of preferentiality and partner selection at the sector level confirm the relevance of international trade costs still today, inducing countries to seek the highest efficiency in their trade patterns.

## Introduction

The past decades have witnessed a remarkable increase of international trade flows among countries, and the involvement of a large number of new players in international markets [1]. Declining transportation and communication costs and lower trade barriers [2], as well as the spreading of international production networks [3], are commonly acknowledged as the main causes for this increase in economic globalization. These changes have dramatically increased the intricacy of world markets, giving rise to new opportunities but also to potentially higher search costs both in industries producing complex manufactured goods with a high technological content and in traditional manufacturing sectors where the number of potential competitors rose rapidly. As a consequence, even in the present integrated world market, firms and countries must compare carefully the costs and benefits of the new opportunities emerging in the world market when seeking to enter in a new foreign market or looking for new suppliers [4, 5].

In this work we apply methods from complex network research to the analysis of world trade at the sector level, in order to unfold the sector components of the world trade network and map specific trade relations among countries. The focus of this work is on discovering patterns of preferentiality in trade flows within a given industry, highlighting possibly different outcomes due to the peculiar characteristics of the industries.

The number of contributions addressing international trade issues from a complex network perspective has been growing in the last years [6–8], but there are still few analyses at the sector level. In the existing works, a common trait about the trade system can be found: centralization and heterogeneous distribution of links that result in hierarchical structures, where the most central countries are often advanced economies or growing giant economies like China. Others recent contributions highlight the strong core/periphery profile of trade relations [9]. Another area of research looks for community or modular structures within the world trade network, eventually producing mixed evidence on the actual relevance of such structures [10–12]. The lack (or very weak evidence) of significant communities at the level of aggregate trade does not mean that such communities do not exist at the industry level. Quite the contrary, the presence of international production ties, or the search for demand in specific markets in a given industry, could give rise to sectoral communities.

The empirical research in international trade has focused mainly on explaining trade pattern among groups of countries by level of GDP, technological development, geographical positioning or participation to preferential trade agreements [13, 14]. The parameters often used to classify countries into groups of preference therefore are related either to membership in a trade agreement or co-location in a geographical region, or consider similarity of countries in terms of some individual economic characteristics (e.g., the level of GDP per capita). Generally speaking, this choice is induced by the expectation that “membership” or “closeness” could create an incentive to trade more. But this is only part of the story. A different perspective suggests that a country could choose to trade more with partners that are “attractive” for other reasons, and because of the existence of tight trade relations, the countries sign a trade agreement, or even see a transformation of their economies [15]. The direction of causality is by no means obvious a priori [16]. Our main aim here is to discover patterns of trade that are characterized by a relatively high degree of “revealed” preferentiality, by means of methods of complex network analysis.

The use of communities detection techniques gives us the opportunity of revealing endogenous clusters of countries with the propensity of concentrating their sectoral trade toward a few partners, taking into account the direction of trade links. In this way we discover groups of countries with relatively more intensive trade relations without imposing any type of pre-determined partition to them, confirming the non-randomness and high preferentiality of the existing trade links.

## Methods

### Communities and pseudocommunities

**Fig 1:**
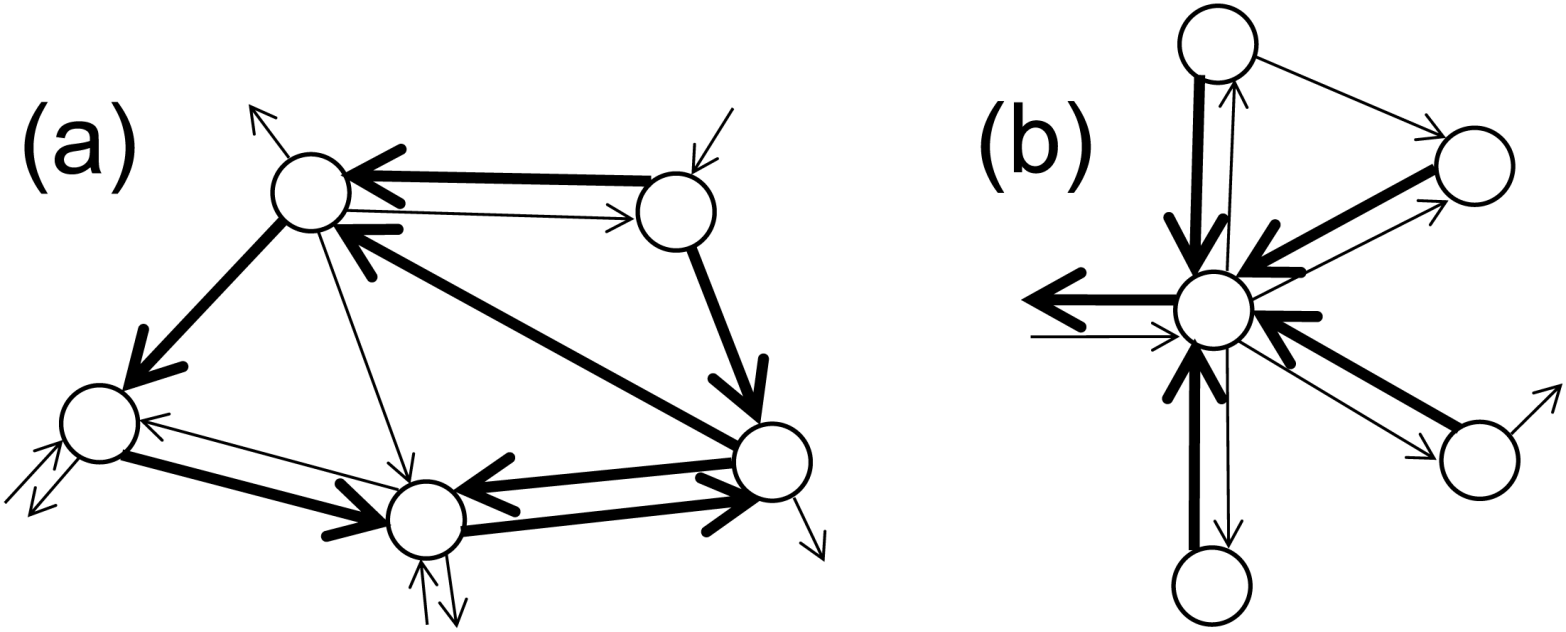
Communities and pseudocommunities. (a) In an *out-community*, all countries export much more within the subnetwork itself than outside. (b) In an *out-pseudocommunity*, most of the countries direct most of their export flow within the subnetwork, but a few of them mainly direct their export to the outside. Examples of *in-community* and *in-pseudocommunity* are obtained by simply reversing the link directions. In the figure, bold links should be interpreted as having much larger weights than thin ones.

In order to detect significant modular structures (*communities*, according to the jargon of network analysis), we adopt the approach recently proposed by [17], which fully takes into account the directionality of links when searching for subnetworks with strong internal connectivity. Consider, for instance, a group of countries which export much more within the group itself rather than outside. Due to their preferential partnership, we would tend to classify them as a community. Yet, we cannot exclude that the same group of countries have large import flows from the outside, which is in contrast with the idea of a community as a set secluded from the rest of the network. This calls for distinguishing between the notion of *out-* and *in-community*: the former is a subnetwork whose countries direct most of their export to within the community rather than to the rest of the network, whereas the latter is such that countries receive most of their import from within the community rather than from the outside. A subnetwork with both features will be denoted as *in/out-community* (Fig 1a).

Another notion relevant to our work is that of *pseudocommunity* [17]. Several real-world networks contain peculiar structures, namely “star-like” subnetworks in which most of the nodes direct most of their out-strength within the subnetwork (often towards a single “central” node), but a few of them (often the “central” node only) mainly direct their out-strength to the outside, so that a subnetwork like this cannot be qualified as a community. Yet, such a structure is worth to be revealed and classified, since it has a special form of strong intra-connectivity: we will denote it as *out-pseudocommunity* (Fig 1b). Dually, an *in-pseudocommunity* will be a subnetwork where most of the nodes receive most of their in-strength from the community rather than from the rest of the network, but a few nodes have instead a large in-strength from the outside. A subnetwork with both features will be denoted as *in/out-pseudocommunity*. As we shall see, in our study we will typically encounter pseudocommunities in sectoral trade flows more often than communities.

We model the trading system as a directed, weighted network with nodes *N* = {1, 2, …, *n*} and weight matrix *W* = [*w*_*ij*_], namely *w*_*ij*_ ≥ 0 is the value of the trade flow from country *i* to country *j*, $s_{i}^{in}=\sum_{j}w_{ji}$ and $s_{i}^{out}=\sum_{j}w_{ij}$ are, respectively, the in- and out-strength of node *i*, and $s_{i}=s_{i}^{in}+s_{i}^{out}$ is the (total) strength. Moreover, we denote by *k*_*i*_ the (total) degree of node *i*, which represents, in our case, the number of import/export trade partners of country *i*.

We denote by *S* the subnetwork induced by a subset *N*_*S*_ ⊂ *N* of the nodes of the original network. Subnetworks are candidates to be communities or pseudocommunities, so that we need a set of suitable indicators to quantify their features. The *persistence probability* *α*_*S*_ of the subnetwork *S* is defined as

$$\alpha_{S}=\sum_{i\in N_{S}}\frac{\pi_{i}}{\Pi_{S}}\sum_{j\in N_{S}}\frac{w_{ij}}{s_{i}^{out}},$$ (1)

where *π*_*i*_ is the so-called PageRank centrality of node *i* (e.g., [18, 19]), and Π_*S*_ = ∑_*i* ∈ *N*_*S*__ *π*_*i*_ is its aggregate value over *S*. Formally, it can be shown [20] that *α*_*S*_ is the probability that a random walker, which is in any of the nodes of *S* at step *t*, remains in *S* at step *t* + 1 (the expected escape time from *S* is thus (1 − *α*_*S*_)^−1^). It is therefore a measure of cohesiveness of *S* and indeed it proved to be an effective tool for the structural analysis of networks [9, 17, 20].

As it is apparent from Eq (1), *α*_*S*_ is a weighted mean of the fraction $w_{ij}/s_{i}^{out}$ of the out-strength of the nodes *i* ∈ *S* which is directed within *S* itself, the weight of the term *i* being the (normalized) centrality *π*_*i*_. Thus, in a trade network, *α*_*S*_ is a weighted mean of the relative export flows that the countries of *S* direct within *S* itself. From Eq (1), it straightforwardly follows that $\alpha_{S}\geq \bar{\alpha }$ when all countries of *S* direct at least a fraction $\bar{\alpha }$ of their export within *S*, so that *out-communities* will be characterized by large values of *α*_*S*_.

Measuring *α*_*S*_ alone may fail in revealing some interesting structures. Consider the subnetwork *S* of Fig 1b: *α*_*S*_ will presumably be small, since a large out-flow from the central node exists. Yet, this structure is frequent in many real-world networks [17], including trade networks, and is worth to be revealed. For that, we define the *average internal strength* *β*_*S*_:

$$\beta_{S}=\sum_{i\in N_{S}}\frac{1}{|N_{S}|}\sum_{j\in N_{S}}\frac{w_{ij}}{s_{i}^{out}},$$ (2)

where ∣*N*_*S*_∣ is the number of nodes of *S*. The quantity 0 ≤ *β*_*S*_ ≤ 1 is simply the arithmetic mean, over the nodes of *S*, of the fraction of the out-strength directed internally to *S* (we recall that *α*_*S*_ is instead a weighted mean of the same quantities). Thus *β*_*S*_ will be large when most of the nodes of *S* direct most of their out-strength within *S*, although a few others could do the opposite yielding a small *α*_*S*_: this is indeed the case of Fig 1b. Notice that, in terms of trade, *β*_*S*_ can be interpreted as the average export share within *S* of the countries of *S*. We define *out-pseudocommunity* a subnetwork with small *α*_*S*_ but large *β*_*S*_. Indeed, it is not a community in the usual sense (i.e., with strong intra- and weak inter-connectivity) but it has nonetheless a special form of strong intra-connectivity, as most of the nodes have their most important connections inside the subnetwork rather than outside.

Having quantified the *out*-properties of *S* by *α*_*S*_ and *β*_*S*_, we need to dually quantify the *in*-properties, i.e., to what extent the *import* flows of the countries of *S* come preferentially from *S*. The most natural way is to define two indicators $\alpha_{S}^{\prime }$ and $\beta_{S}^{\prime }$ by simply reversing the direction of each link in the original network (this actually corresponds to consider the network defined by the transpose weight matrix *W*′ = *W* ^*T*^), and then define the new indicators by using, on this new network, the same definitions used above for *α*_*S*_ and *β*_*S*_ (see [17] for details). We obtain:

$$\alpha_{S}^{\prime }=\sum_{i\in N_{S}}\frac{\pi_{i}^{\prime }}{\Pi_{S}^{\prime }}\sum_{j\in N_{S}}\frac{w_{ji}}{s_{i}^{in}},\;\beta_{S}^{\prime }=\sum_{i\in N_{S}}\frac{1}{|N_{S}|}\sum_{j\in N_{S}}\frac{w_{ji}}{s_{i}^{in}},$$ (3)

where now $\pi_{i}^{\prime }$ is the PageRank centrality of the network with weight matrix *W*′. Obviously the subnetwork *S* should be characterized at the same time by both its in- and out-attributes. To get a complete picture, thus, we have to associate to *S* the full set $\left(\alpha_{S},\beta_{S},\alpha_{S}^{\prime },\beta_{S}^{\prime }\right)$ of four indicators, and assess whether one or more of them are large enough to reveal that *S* has the above described in- and/or out-properties. In this work, we adopt the value 0.5 as a threshold of significance for the above four indicators—roughly speaking, values larger than 0.5 mean that the countries of *S* prefer to trade with other members of *S* for at least half of value (we note that this is a generalization of the notion of community as defined by [21]). Therefore, by combining the in- and out-properties above discussed, we arrive at the definition of 8 possible types of subnetworks (*structures*) of interest for (pseudo)community analysis, which are summarized in Table 1. Each one of them corresponds to a specific combination of in-/out- as well as intra-/interconnectivity.

**Table 1:** The eight types of structures of interest for (pseudo)community analysis in directed networks.

	*α*_*S*_	*β*_*S*_	$\alpha_{S}^{\prime }$	$\beta_{S}^{\prime }$	*ϕ*_*S*_
**OC**: out-community	**≥ 0.5**	**≥ 0.5**	–	*< 0.5*	$\max\{1-\alpha_{S},1-\beta_{S},\beta_{S}^{\prime }\}$
**IC**: in-community	–	*< 0.5*	**≥ 0.5**	**≥ 0.5**	$\max\{\beta_{S},1-\alpha_{S}^{\prime },1-\beta_{S}^{\prime }\}$
**IOC**: in/out-community	**≥ 0.5**	**≥ 0.5**	**≥ 0.5**	**≥ 0.5**	$\max\{1-\alpha_{S},1-\beta_{S},1-\alpha_{S}^{\prime },1-\beta_{S}^{\prime }\}$
**OP**: out-pseudocommunity	*< 0.5*	**≥ 0.5**	–	*< 0.5*	$\max\{\alpha_{S},1-\beta_{S},\beta_{S}^{\prime }\}$
**IP**: in-pseudocommunity	–	*< 0.5*	*< 0.5*	**≥ 0.5**	$\max\{\beta_{S},\alpha_{S}^{\prime },1-\beta_{S}^{\prime }\}$
**IOP**: in/out-pseudocommunity	*< 0.5*	**≥ 0.5**	*< 0.5*	**≥ 0.5**	$\max\{\alpha_{S},1-\beta_{S},\alpha_{S}^{\prime },1-\beta_{S}^{\prime }\}$
**IPOC**: in-pseudocom./out-com.	**≥ 0.5**	**≥ 0.5**	*< 0.5*	**≥ 0.5**	$\max\{1-\alpha_{S},1-\beta_{S},\alpha_{S}^{\prime },1-\beta_{S}^{\prime }\}$
**ICOP**: in-com./out-pseudocom.	*< 0.5*	**≥ 0.5**	**≥ 0.5**	**≥ 0.5**	$\max\{\alpha_{S},1-\beta_{S},1-\alpha_{S}^{\prime },1-\beta_{S}^{\prime }\}$

For each type of structure, the subnetwork *S* is considered significant if the constraints on $\left(\alpha_{S},\beta_{S},\alpha_{S}^{\prime },\beta_{S}^{\prime }\right)$ specified in the table are fulfilled: *ϕ*_*S*_ quantifies the distance of *S* from ideality.

For each type of structure, we can summarize the connectivity properties of the subnetwork *S* by means of a scalar indicator *ϕ*_*S*_ which depends on $\left(\alpha_{S},\beta_{S},\alpha_{S}^{\prime },\beta_{S}^{\prime }\right)$ and quantifies the distance of *S* (in infinite norm) from the ideality of that type of structure. For instance (see Table 1), the ideal in-pseudocommunity/out-community (IPOC) must have large *α*_*S*_ to be qualified as out-community, as discussed above, which also implies large *β*_*S*_. It also must have large $\beta_{S}^{\prime }$ to be qualified as in-pseudocommunity, but small $\alpha_{S}^{\prime }$, otherwise it would be an in-community too. Thus $\left(\alpha_{S},\beta_{S},\alpha_{S}^{\prime },\beta_{S}^{\prime }\right)$ should ideally tend to (1, 1, 0, 1): *ϕ*_*S*_ is the distance from this point, and the smaller is *ϕ*_*S*_, the more the structure is significant.

### Local (pseudo)community search

A number of local methods for community analysis have been proposed in recent years (see, e.g., [22, 23] for early contributions). Here we use the algorithm described in [17], which is here briefly described.

We start from a single node *k*, so that the initial current subnetwork is *S*_*k*_ = {*k*} and *ϕ*_*S*_*k*__ = *ϕ*_*k*_ = 1 (note that *ϕ*_*S*_ is defined according to the type of structure we are seeking for, see last column of Table 1). At each step, we include into *S*_*k*_ the node, selected among those neighboring *S*_*k*_, that attains the maximal decrease of *ϕ*_*k*_. We stop when we get a local minimum for *ϕ*_*k*_, namely when any new node insertion would increase *ϕ*_*k*_. More precisely, to filter out possible small fluctuations of *ϕ*_*k*_, we stop when *ϕ*_*k*_ increases of at least *r* = 0.025 if a new node is introduced (this value has been tuned by trial-and-error). The (pseudo)community *S*_*k*_ is the subnetwork which attains the minimum of *ϕ*_*k*_: it is retained only if *ϕ*_*k*_ < 0.5, consistently with Table 1, otherwise it is discarded.

The procedure is repeated for each starting node *k* = 1, 2, …, *N*, yielding the set **S** = {*S*_1_, *S*_2_, …} of (pseudo)communities. Notice that not necessarily a valid (pseudo)community *S*_*k*_ is found from any starting node *k*, because we could find no minima for *ϕ*_*k*_ as *S*_*k*_ grows to the entire network, or the minimum could have *ϕ*_*k*_ ≥ 0.5 denoting a non significant (pseudo)community.

**Fig 2:**
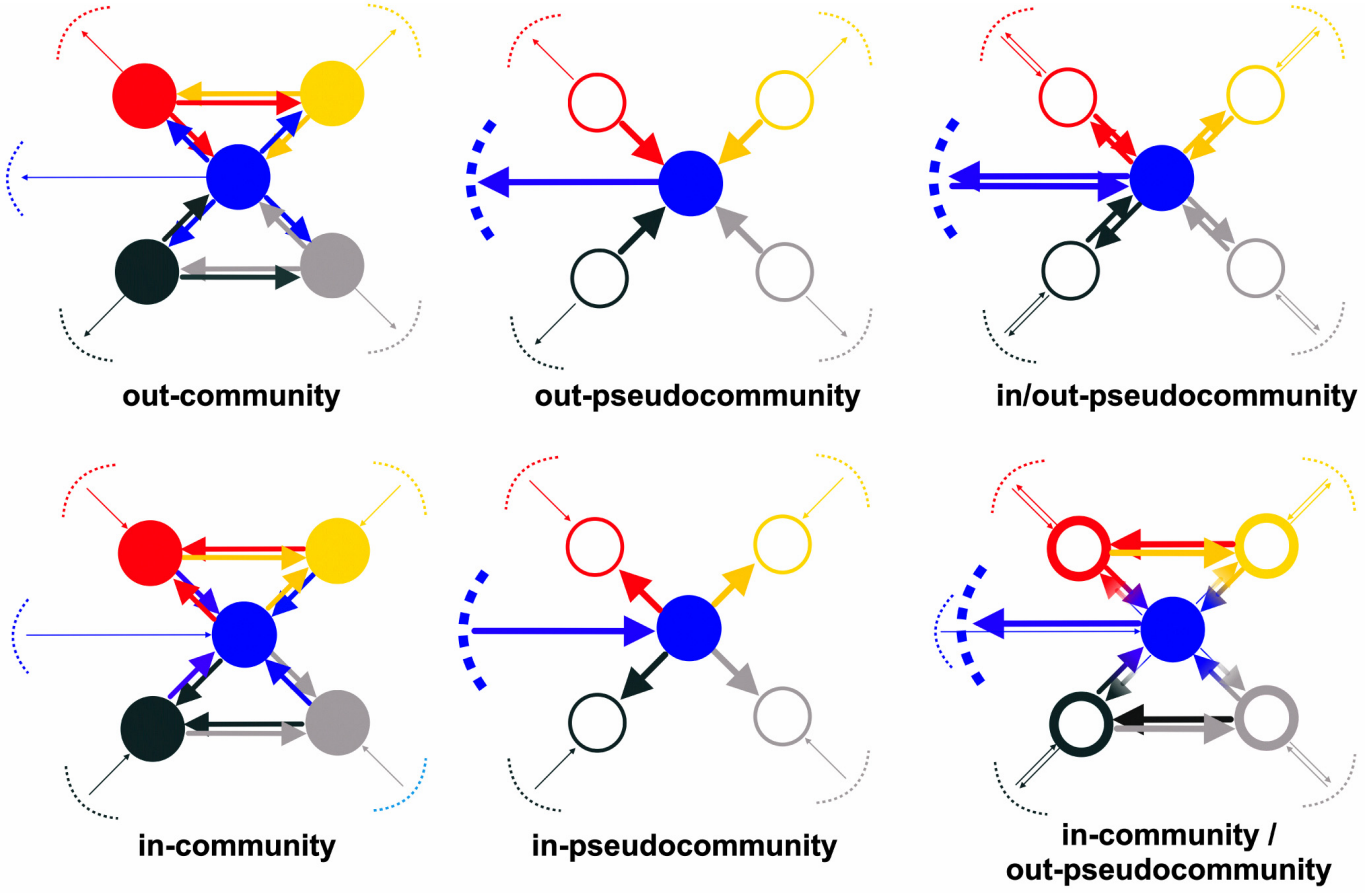
A pictorial representation of the six types of structures found in the sectoral world trade dataset. The link arrows denote the direction of traded goods. Bold links are those with comparatively large trade flows. Link color refers to the country in respect to which the trade flow is normalized (see Eqs (1)–(3)). Dashed lines represent Rest-of-World (RoW), with thickness denoting the relative importance as (aggregate) trade partner.

Fig 2 provides a pictorial representation of the six types of structures (over eight) that have actually been found in our dataset (see section Results). As a matter of fact, not all theoretical structures have the same probability to occur in international trade, as they describe different economic organizations. In- and out-communities represent groups of countries with large internal trade: however, whereas in-communities have small import flows from the Rest-of-World (RoW), they may display large export flows toward it, and the opposite holds for out-communities. The isolation from RoW of pseudo-communities is lower thanks to (at least) one country in the structure importing a lot from RoW (in-pseudocommunity) or exporting a lot outside of the structure (out-pseudocommunity), while the other countries are only indirectly connected to the RoW in terms of imports or exports. The first case can occur for a group of countries lacking the appropriate infrastructures to receive large import flows of some kind, while the second case can occur for example when we have a group of small countries unable to reach distant foreign markets by themselves, thus using a larger country as an exporting hub. The remaining structures in the picture are combinations of the ones described above.

## Results

### Sectoral trade networks: Textiles and Electronics

We focus our investigation on international trade flows of *Textiles and Textile Articles* (from now on simply Textiles, for short) and *Electronics*, two categories of manufactured goods identified in the Harmonized System (HS) classification. The first category corresponds to the sector codes from 50 to 63 of Section XI of HS 2002 Classification, while the second one corresponds to code 85 within Section XVI. For more details about the Sections breakdown see [24]. We choose these two sectors because they are representative of, respectively, a traditional manufacturing sector, with production capabilities virtually in all countries, and a high-tech sector that requires a much more sophisticated production technology and with a differentiated diffusion among consumers worldwide. These two sectors are indeed classified as low-technology and high-technology, respectively, by the OECD classification [25]. In principle, therefore, production and consumption of these two types of goods are likely to give rise to different patterns of international trade.

For the two sectors, data on bilateral trade among 221 countries were collected from CEPII-BACI database [26], drawing on the UN Comtrade database [27] (the use of this database to perform network analysis at the sector level is presented in [28]) for the year 2006, chosen to avoid possible disruptions generated by the international financial crisis, and implementing the HS Classification. This dataset includes in principle directed trade flows as declared by each exporting country to each of its destination market, as well as trade flows declared by each importing country received from each of its suppliers. Therefore data are available in two matrices that should be one the transposition of the other, one placing exporters as reporting countries in rows and importers (partner countries) in column, and viceversa in the other, but obviously maintaining the flow direction. In reality, the two matrices are not exactly one the transposition of the other, because of data collection problems and different declaration rules across countries. The CEPII-BACI database has been cleaned for some these discrepancies, but we chose to use as weights in our analysis the matrix of declared imports (which can be read as the flows of exports from the point of view of the origin countries), following [8, 29]. According to the CEPII-BACI database, in 2006 trade flows for Textiles and Electronics among all 221 countries amounted to about 543 billion and 1600 billion US dollars, respectively, representing 5% and 16% of total world trade of 2006.

**Fig 3:**
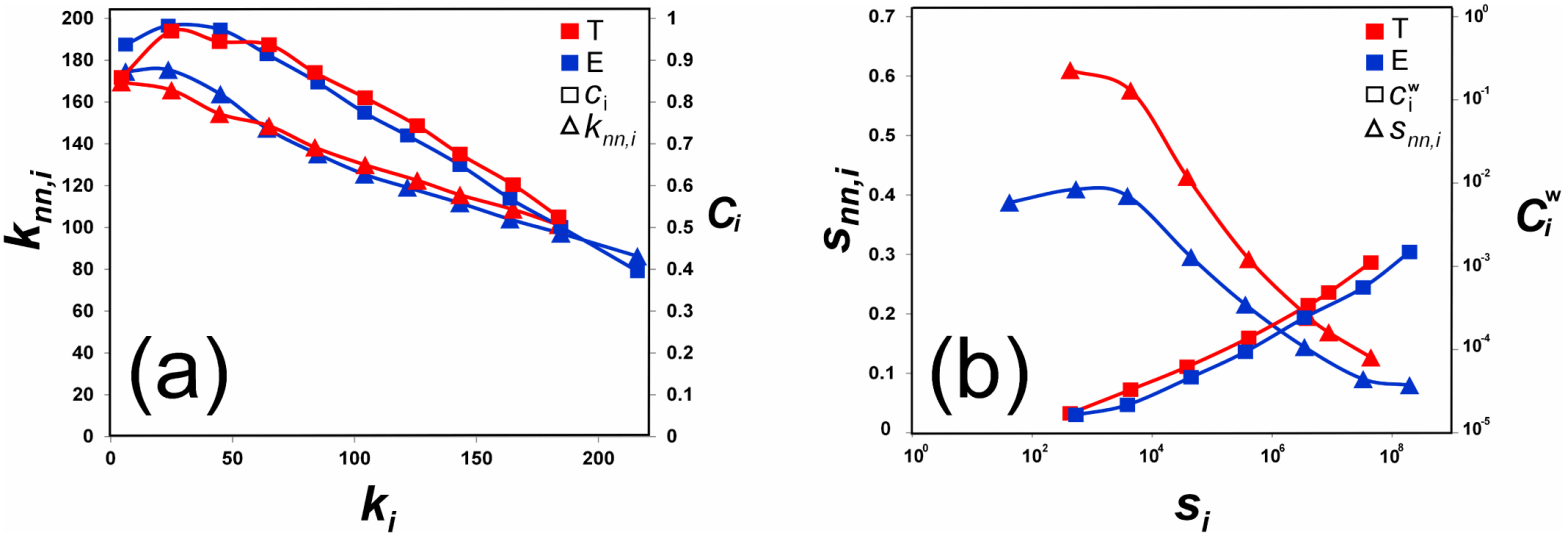
Disassortativity and clustering coefficients in the sectoral trade networks of Textiles (T) and Electronics (E). (a) The average nearest neighbor degree *k*_*nn*,*i*_ (left scale) and the clustering coefficient *c*_*i*_ (right scale) of node *i*, as a function of the degree *k*_*i*_. (b) The average nearest neighbor strength *s*_*nn*,*i*_ (left scale), normalized by max_*i*_ *s*_*nn*,*i*_, and the weighted clustering coefficient $c_{i}^{w}$ (right scale) of node *i*, as a function of the strength *s*_*i*_.

The two sectoral trade networks under scrutiny are connected [18], i.e., there are no disconnected countries or groups of countries. In Table 2 we report a few global indicators, computed on the undirected (symmetrized) networks (i.e., link directions are neglected). Both networks are very dense, similarly to the total (i.e., all sectors) world trade network (e.g., [8, 12]), and characterized by a high level of average local clustering (we recall that, in an undirected network, the density is the fraction of existing links *L* with respect to their maximum allowed number, which is *N*(*N* − 1)/2. The clustering coefficient is the average, over all *N* nodes, of the fraction of triangles *t*_*i*_ attached to node *i* with respect to their maximum allowed number *k*_*i*_(*k*_*i*_ − 1)/2, where *k*_*i*_ is the degree of node *i* [18, 19]). They also display quite large degree disassortativity (Fig 3a), meaning that countries with few trading partners tend to connect to those countries which have instead many partners, and viceversa (we recall that the degree/strength assortativity is measured by the Pearson correlation coefficient between the degrees/strengths of the nodes connected by all the *L* network links [18, 19]). Disassortativity also holds in terms of strength, although to a lesser extent (Fig 3b).

**Table 2:** Network-based indicators of the two analyzed sectoral trade networks.

	density	clustering coefficient	degree assortativity	strength assortativity
Textiles	0.620	0.819	-0.387	-0.085
Electronics	0.605	0.820	-0.434	-0.114

All indicators are computed on the undirected (symmetrized) networks (i.e., link directions are neglected).

As shown in Fig 3a, countries with low degree tend to have large clustering coefficient, meaning that the few partners they trade with are, with large probability, connected each other. This is not surprising, since those latter countries are typically the hubs of the network, namely the most important (and thus connected) countries. The situation is, however, different if we look at the *weighted* clustering coefficient $c_{i}^{w}$ which, for node *i*, is obtained by replacing the number of triangles *t*_*i*_ in the unweighted clustering coefficient formula by the sum of the *triangle intensities*, namely the cube root of the product of the weights of the triangle links [30] (weights have to be previously normalized by the maximum network weight, to avoid scale effect). In this way we measure the “intensity” of the trade relationships surrounding a country and, as expected, this indicator turns out to be increasing with the strength of the country, meaning that countries with smaller strength are typically included in triangles with smaller intensity (Fig 3b).

### (Pseudo)community analysis

We applied the above described local searching algorithm to sectoral trade data in Textiles and Electronics, and we found evidence of the existence of significant subnetworks in which preferential trade occurs. The observed structures, which are summarized in Table 3, are “clustered” groups of countries that trade goods for consumption and production within a specific sector.

**Table 3:** Number and size of the detected (pseudo)communities.

	**Textiles**		**Electronics**	
	number	size	number	size
**OC**: out-community	–	–	5	16.2(14–17)
**IC**: in-community	–	–	2	35.0(26–44)
**OP**: out-pseudocommunity	119	20.4(2–39)	84	17.6(3–41)
**IP**: in-pseudocommunity	44	15.8(2–27)	47	28.4(4–45)
**IOP**: in/out-pseudocommunity	38	22.7(9–35)	35	21.8(15–27)
**ICOP**: in-com./out-pseudocom.	5	20.2(17–25)	–	–

For each type of structure (OC, IC, etc.) and for each sector (Textiles, Electronics), the table reports the number of (pseudo)communities detected as specified in the section Methods, as well as their size (mean (minimum–maximum) number of countries).

Table 3 highlights the predominance of pseudocommunities of different kind (in-, out-, and in-/out-pseudocommunities) in organizing the patterns of preferential trade. A few significant in- and out-communities have also been found but, overall, pseudocommunities are much more numerous. This result is in line with the increased interdependence of countries, making it much more difficult to find independent and isolated groups of countries, which we instead observed more easily in the past mostly for political rather than economic reasons. In fact, no in-/out-community has been detected, pointing out that there are no groups of countries which are self-sufficient both on the import and on the export side.

To better interpret our results, we checked for the existence of similar structures using data for different years, finding indeed that similar structures can be observed also at different points in time, and they are not a peculiarity of the year we picked. In particular, if comparing our results with a decade earlier (1995), we see that the number of structures decreases over time, and their composition changes remarkably. While the central countries in these structures (as defined below) are relatively stable, their partners vary as the number of active traders in the world market, especially among less developed countries, grows. (Results on structures’ composition in different years are available from the authors upon request). We interpret these changes as evidence of an evolution of the world trading system—at least in the sectors examined—to include new and more connected nodes, and having fewer closed structures, what is commonly indicated with the term ‘globalization’.

To be more specific, from Table 3 we note that no significant in- or out-communities are observed in a traditional sector such as Textiles, whose production and use are very common in virtually all countries. By contrast, in the Electronics sector, where technological barriers give rise to selection of exporters and markets, we do find communities, as well as a much larger number of out-pseudocommunities.

To measure the relevance of the detected structures in shaping sectoral world trade, Table 4 reports, for each of the two sectors, the share of trade within all (pseudo)communities of a given structure type, with respect to world sectoral trade (column *internal/world trade*). In many instances, the value of such a share proves that a significant portion of trade takes place within specifically organized structures of countries. Even more noticeable, the table also reports the share of trade within all (pseudo)communities of a given structure type, with respect to the total sectoral trade of its member countries (column *internal/total trade*). It turns out that preferentiality affects an important proportion of sectoral world trade and, more importantly, for all those countries that are directly involved into these structures, preferential trade constitutes the majority of their trade (see also the last row “all structures”). The higher preferentiality of trade in Electronics emerges also from the fact that the share of trade occurring within communities and pseudocommunities is, in almost all instances, higher in this industry than in Textiles.

**Table 4:** Share of sectoral trade in the detected (pseudo)communities.

	**Textiles**		**Electronics**	
	internal/world trade (%)	internal/total trade (%)	internal/world trade (%)	internal/total trade (%)
**OC**: out-community	–	–	0.67	51.8
**IC**: in-community	–	–	22.1	67.9
**OP**: out-pseudocommunity	4.70	42.9	6.77	32.9
**IP**: in-pseudocommunity	8.85	27.7	10.5	29.7
**IOP**: in/out-pseudocommunity	10.4	30.5	13.3	35.3
**ICOP**: in-com./out-pseudocom.	25.0	51.7	–	–
all structures	85.5	85.4	87.2	85.3

The table reports, for each sector, the share of trade within the set of countries included in all (pseudo)communities of a given structure type, with respect to world sectoral trade (column *internal/world trade*), and with respect to the total sectoral trade of its member countries (column *internal/total trade*). Last row (“all structures”) considers the trade flows included in at least one structure.

It is not surprising to discover that, within a structure, one or a few nodes play a central role, receiving relatively more preferences as destination market or source of supply. Asymmetries in the position of nodes were spotted across all the significant structures we found, suggesting that preference in trade is driven by those few countries chosen by many other countries as the main trade partners. To discuss this aspect, we now take a closer look at two examples, an out-pseudocommunity in Textiles and an in-community in Electronics. Both subnetworks are characterized by a strongly heterogeneous, core-periphery pattern, with a majority of peripheral countries almost exclusively connected to the core countries, which in turn are connected to almost all the members. Put it differently, in spite of the increased involvement of emerging and developing countries in world trade, still only a few countries have a relevant role as markets of destination or sources of supply of goods.

**Fig 4:**
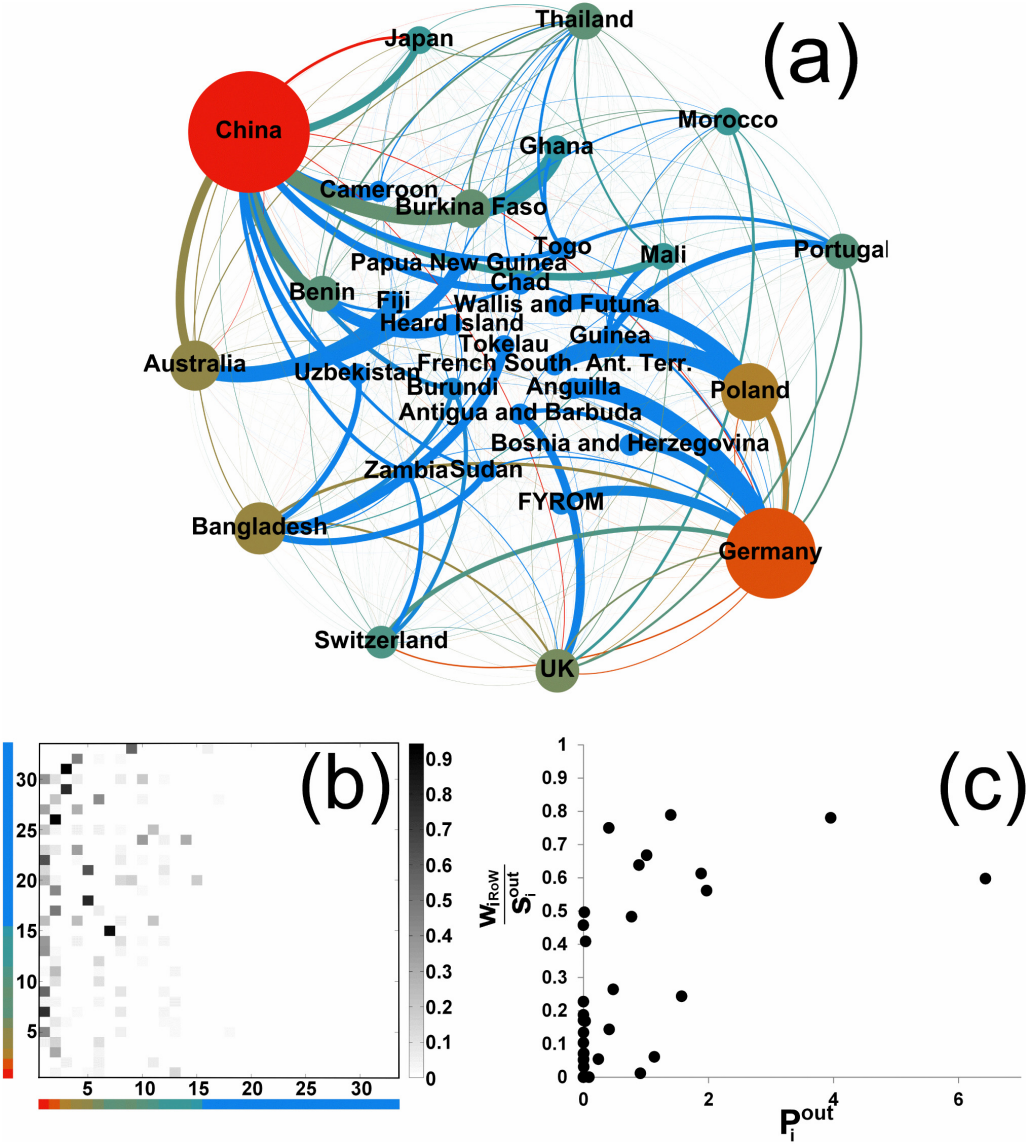
An example of out-pseudocommunity in Textiles. (a) In the graph representation, the color and size of nodes is related to the preference centrality $P_{i}^{out}$ (Eq (4)), and the thickness of links is proportional to the normalized export flows of source nodes ($w_{ij}/s_{i}^{out}$). (b) Preference matrix: the 33 countries of the out-pseudocommunity have been reordered by their $P_{i}^{out}$ value. Color bars refer to color nodes in panel (a). The (*i*, *j*) entry is $w_{ij}/s_{i}^{out}$, i.e., the row-normalized flow from row country *i* to column country *j*. The matrix pattern, with the empty right-hand part, reveals the core-periphery structure of the subnetwork. (c) The export share of each country of the out-pseudocommunity towards the Rest-of-World (RoW) versus its preference centrality $P_{i}^{out}$.

In the case of the *out-pseudocommunity* in Textiles of Fig 4a, trade occurring within this structure amounts at about 12% of world trade and about 28% of total trade of member countries. Germany and China are the dominant countries, and what qualifies this subnetwork as an out-pseudocommunity is that these two countries export mainly to the outside. We can quantify the role, within the structure, of a given country *i* by measuring the preference in trade (export, in this case) given to that country by all the other members of the subnetwork:

$$P_{i}^{out}=\sum_{j\in N_{S}}\frac{w_{ji}}{s_{j}^{out}}.$$ (4)

The quantity $P_{i}^{out}$, that we denote as *preference centrality*, captures the fact that the majority of the members of the subnetwork export mainly towards few of them only. Indeed, Fig 4c highlights the inhomogeneity in the $P_{i}^{out}$ values within the structure, with the few countries with large $P_{i}^{out}$ having an important share trade to the Rest-of-World (RoW).

**Fig 5:**
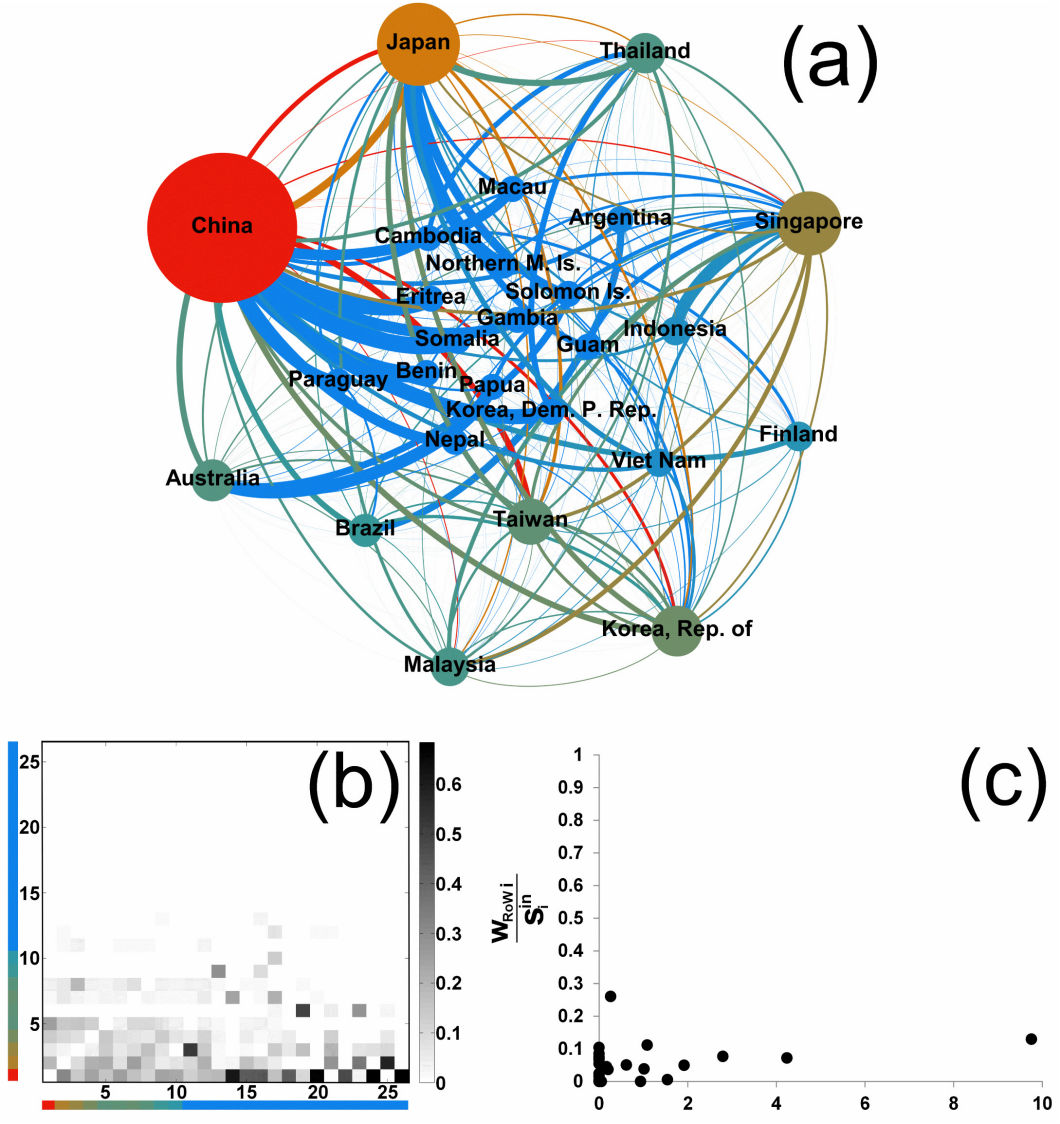
An example of in-community in Electronics. (a) In the graph representation, the color and size of nodes is related to the preference centrality $P_{i}^{in}$ (Eq (5), and the thickness of links is proportional to the normalized import flows of target nodes ($w_{ij}/s_{j}^{in}$). (b) Preference matrix: the 26 countries of the in-community have been reordered by their $P_{i}^{in}$ value. The matrix pattern, with the empty upper part, reveals the core-periphery structure of the subnetwork. Color bars refer to color nodes in panel (a). The (*i*, *j*) entry is $w_{ij}/s_{j}^{in}$, i.e., the column-normalized flow to column country *j* from row country *i*. (c) The import share of each country of the in-community from the Rest-of-World (RoW) versus its preference centrality $P_{i}^{in}$.

The *in-community* of Fig 5 is instead organized around China and Japan, which are indeed the most relevant sources of supply for the rest of the subnetwork. Here we quantify the preference centrality by

$$P_{i}^{in}=\sum_{j\in N_{S}}\frac{w_{ij}}{s_{j}^{in}},$$ (5)

so that countries with larger $P_{i}^{in}$ are those from which the members of the subnetwork import systematically more. Being an in-community means that also central nodes mainly import from within the subnetwork: this is testified by Fig 5c, which shows that none of the countries has a large import share from the Rest-of-World. As a matter of fact, the import trade occurring within the structure in respect to the total trade of the group members is around 70%. This import value also represents about 22% of world trade. It is worth noticing that the classification of this subnetwork as an in-community implies that it must have a significant export flow towards the Rest-of-World (otherwise it would have been qualified as in-/out-communities): indeed, it turns out that 55% of the total export is directed outside the community.

### A measure of (pseudo)community centrality

As we already pointed out, the (pseudo)communities detected in the sectoral trade networks have a core-periphery (or star-shaped) structure, which is worthwhile to be explored in order to rank countries in terms of their centrality within such structures. Besides the preference centrality $P_{i}^{in/out}$ above discussed, we introduce another measure of the importance of country *i* based on the sensitivity of the (pseudo)community to variations of its composition, more precisely to the removal of node *i*. A similar method has been used in [31] to detect core nodes within community structures.

We quantify the significance of the subnetwork *S* by the indicator *ϕ*_*S*_ discussed in the section Methods (see Table 1). The more a country *i* is central, the more sensitive *ϕ*_*S*_ will be to the variation in the (pseudo)community composition consisting in the removal of *i* from *S*: thus the centrality $C_{i}^{S}$ of node *i* belonging to the (pseudo)community *S* is defined as

$$C_{i}^{S}=\frac{|\phi_{S}-\phi_{S-\{i\}}|}{\left\langle \right|\phi_{S}-\phi_{S-\{j\}}{\left|\right\rangle }_{j\in N_{S}}},$$ (6)

namely as the variation of *ϕ*_*S*_ due to the removal of *i*, normalized by the average of such a quantity over all nodes of *S*. Notice that the centrality $C_{i}^{S}$ of country *i* is a function of the (pseudo)community *S* which is considered. Typically, however, *i* is assigned to many different overlapping (pseudo)communities by the local search algorithm. We thus characterize country *i* by its average community centrality $<C_{i}^{S}>$ computed over all the (pseudo)communities *S* of a given type (e.g., out-communities) *i* belongs to.

**Fig 6:**
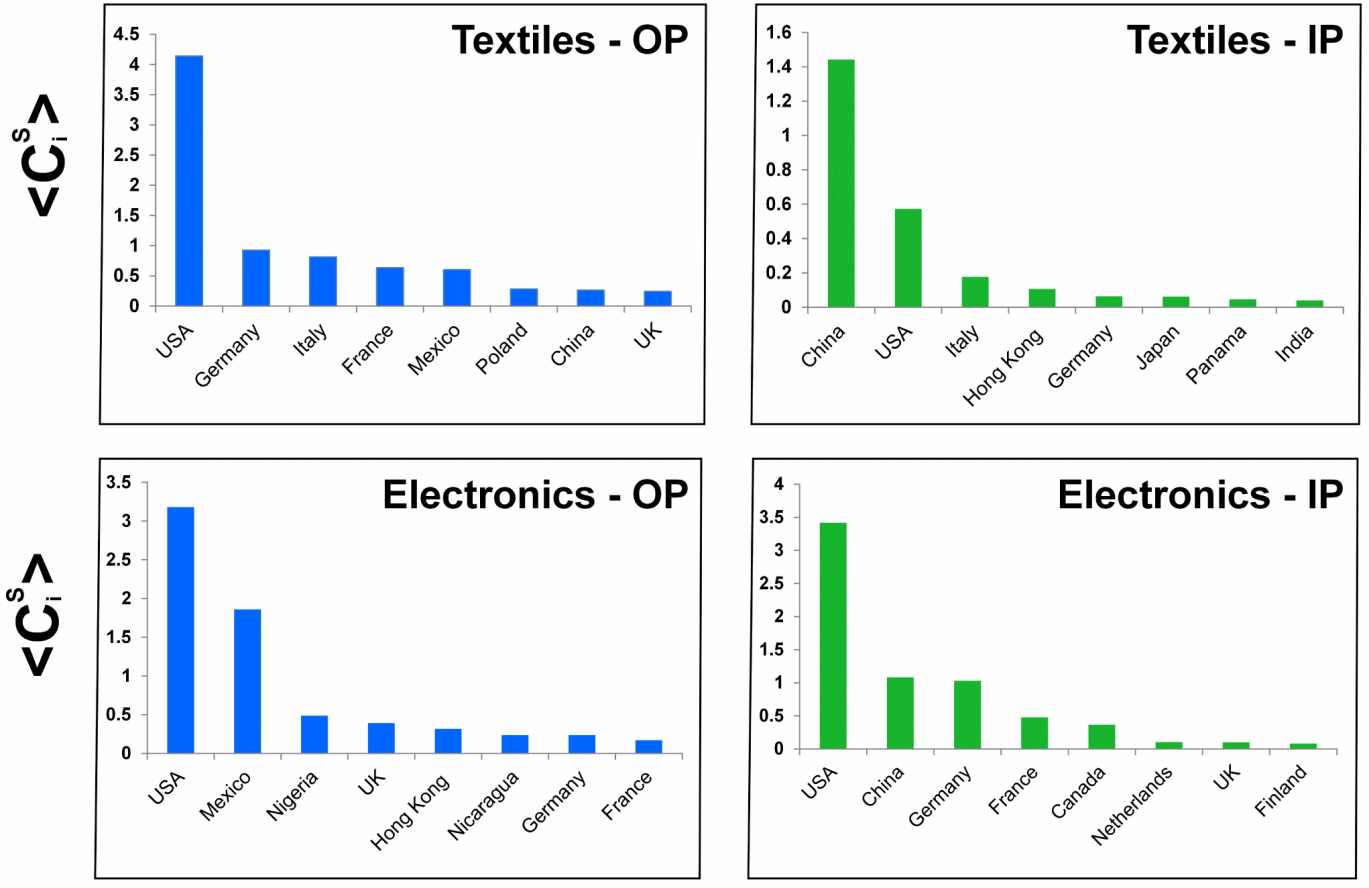
The top countries for (pseudo)community centrality $C_{i}^{S}$ in out-pseudocommunities (left) and in-pseudocommunities (right), for the two sectoral trade networks of Textiles (above) and Electronics (below). The average centrality $<C_{i}^{S}>$ is computed over the set of out- (left panels) or in-pseudocommunities (right panels) country *i* belongs to.

The above defined centrality measure is aimed at emphasizing the role of those countries which are the most relevant markets of destination or sources of supply for most of the others members of the (pseudo)community. Fig 6 shows the top countries, in terms of their average $C_{i}^{S}$, for the set of out- and in-pseudocommunities (OP and IP, resp.) detected in the two sectoral trade networks of Textiles and Electronics (we restrict our analysis to pseudocommunities for brevity). Not surprisingly, countries with the largest trade volumes (e.g., USA, China, Germany, UK) are also systematically those displaying the largest community centrality. China appears to be an important partner for many countries as a supplier both of Textiles and Electronics (see IP panels). The USA play a double role, since they are a market of destination in both sectors (see OP panels), but they are also one of the most relevant sources of supply. Overall, these evidences suggest that bigger countries have an organizing role in preferential structures. The reason is intuitive: preferentiality involve bigger countries because the higher dimension of markets justify consumption goods trade to and from exclusively bigger countries, and endowment of capital and production factors justifies import and export of intermediate inputs for transformation within their national boundaries into final goods. In the sectors examined, it is very rare to find structures that mark an exception to the general rule seeing the largest trading countries at world level as central also in the specific structures. For example, in Electronics, one out-community is formed by a group of former members of the Soviet Union, with Russia (certainly not a large electronics trader at the world level) at the core of such structure: this occurrence can be due for example to the development in the past of different standards in this sector for these countries. In other peculiar sectors, not examined here, such exceptions can be less rare, but in the large important industries analyzed here, the role of bigger countries seems to prevail also in the formation of communities and pseudo-communities.

**Fig 7:**
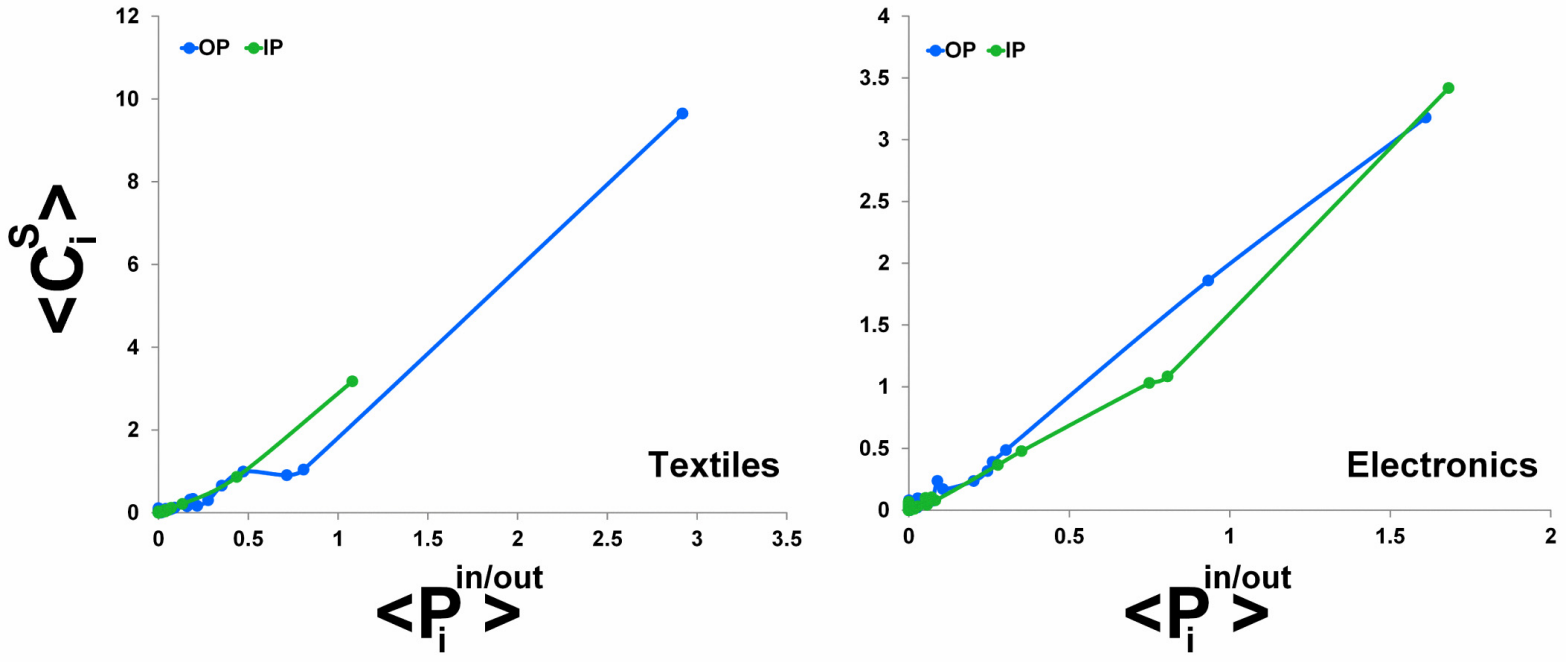
The relationship between the (pseudo)community centrality $C_{i}^{S}$ and the preference centrality $P_{i}^{in/out}$. The average values are computed over the set of pseudocommunities country *i* belongs to.

We finally investigate the relationship between the (pseudo)community centrality $C_{i}^{S}$ and the preference centrality $P_{i}^{in/out}$ previously discussed (both averaged over all structures country *i* belongs to) and, not surprisingly, we discover that the two quantities are roughly proportional (Fig 7). This confirms that those countries which, within (pseudo)communities, are the preferential markets of destination or sources of supply, have also a pivotal role in the structural cohesiveness of the (pseudo)communities.

## Discussion

Applying the tools of network analysis to sectoral trade data reveals groups of countries with a distinct propensity to concentrate their trade among a few partners. We found that preferentiality is a relevant feature of trade within a given industry, i.e., countries generally prefer to concentrate their trade (exports or imports) toward one or a small group of partners, often the most important economies of the world but also the most connected by the system of trade relations. The results indicate that countries do not find it optimal to maximize the number of their connections, but they rather select who to link to, as not every country can offer the same level of access to the global market or the same characteristics of suppliers. This selection process produces as an outcome a strongly asymmetrical organization of international trade, showing sparse and not reciprocating structures, featuring few leading countries, from which many other countries are dependent in exporting and importing goods. These structures have been detected and highlighted by means of the recently proposed notions of in/out-(pseudo)communities, which allows one to precisely unfold the subtle differences in the cohesiveness properties of different subnetworks.

Even if preferentiality characterizes trade relations in both the sectors examined (Textiles and Electronics), the revealed structures are different across industries and goods’ categories. For instance, preferences are strong for importing intermediate inputs in high-tech sectors, as the Electronics case suggests, where inputs can be quite sophisticated. But preferences also arise for exports of finished products, with a few very large markets shaping the trade structure, as shown in a low tech and traditional sector such as Textiles. This evidence can be systematically verified by testing more sectors, but it is already suggestive of important differences existing in the world trade network when considering trade flows at the industry level.
